# QTL Underlying Circadian Clock Parameters Under Seasonally Variable Field Settings in *Arabidopsis thaliana*

**DOI:** 10.1534/g3.118.200770

**Published:** 2019-02-12

**Authors:** Matthew J. Rubin, Marcus T. Brock, Seth J. Davis, Cynthia Weinig

**Affiliations:** * Department of Botany, University of Wyoming, Laramie, WY 82071; † Program in Ecology, University of Wyoming, Laramie, WY 82071; ‡ Department of Biology, University of York, Heslington, York, YO10 5DD, UK; § Department of Molecular Biology, University of Wyoming, Laramie, WY 82071

**Keywords:** *Arabidopsis thaliana*, circadian clock, plant shoot architecture, quantitative trait loci

## Abstract

The circadian clock facilitates coordination of the internal rhythms of an organism to daily environmental conditions, such as the light-dark cycle of one day. Circadian period length (the duration of one endogenous cycle) and phase (the timing of peak activity) exhibit quantitative variation in natural populations. Here, we measured circadian period and phase in June, July and September in three *Arabidopsis thaliana* recombinant inbred line populations. Circadian period and phase were estimated from bioluminescence of a genetic construct between a native circadian clock gene (*COLD CIRCADIAN RHYTHM RNA BINDING 2*) and the reporter gene (*LUCIFERASE*) after lines were entrained under field settings. Using a Bayesian mapping approach, we estimated the median number and effect size of genomic regions (Quantitative Trait Loci, QTL) underlying circadian parameters and the degree to which these regions overlap across months of the growing season. We also tested for QTL associations between the circadian clock and plant morphology. The genetic architecture of circadian phase was largely independent across months, as evidenced by the fact that QTL determining phase values in one month of the growing season were different from those determining phase in a second month. QTL for circadian parameters were shared with both cauline and rosette branching in at least one mapping population. The results provide insights into the QTL architecture of the clock under field settings, and suggest that the circadian clock is highly responsive to changing environments and that selection can act on clock phase in a nuanced manner.

The natural environment is heterogeneous, and organisms experience temporally variable conditions over the course of hours within a day and days within a growing season. Circadian clocks comprise endogenous repeating rhythms that are set through the perception and integration of many environmental factors (Dvornyk *et al.* 2003; Bell-Pedersen *et al.* 2005; Harmer 2009; Edgar *et al.* 2012), and enable coordination of biological activities with the external environment. The molecular basis of the circadian system is a complex network of interconnected feedback loops that have been identified through molecular genetic approaches (Hsu and Harmer 2014). In the past several years, quantitative trait loci regulating circadian traits have been mapped under controlled settings in the model plant *Arabidopsis thaliana* (Swarup *et al.* 1999; Michael *et al.* 2003; Edwards *et al.* 2005; Darrah *et al.* 2006; Boikoglou *et al.* 2011; Anwer *et al.* 2014; De Montaigu *et al.* 2015), *Brassica rapa* (Edwards *et al.* 2011; Lou *et al.* 2011; Xie *et al.* 2015), and cultivated tomato, *Solanum lycopersicum (*Müller *et al.* 2016*)*. Interestingly, the periodicity (duration of a single cycle) and phase (timing of peak activity) of the circadian clock as measured in controlled settings also differ significantly among plant populations (Michael *et al.* 2003; De Montaigu *et al.* 2015) and among genotypes within a population (Salmela *et al.* 2016), indicating segregating variation at clock loci in natural populations. Further, phase of the clock is variable among genotypes raised in field settings and is highly responsive to month of the growing season (Matsuzaki *et al.* 2015; Rubin *et al.* 2017), demonstrating sensitivity of the clock to complex natural environments. Yet, the QTL architecture and environmental sensitivity of quantitative variation in circadian period and phase remains uncharacterized among plants grown under natural field conditions.

Phenotypic outputs of the circadian clock involve diverse processes in plants, including gene expression (Harmer *et al.* 2000; Covington *et al.* 2008; Matsuzaki *et al.* 2015), growth and phenology (reviewed in Greenham and Mcclung 2015; Edwards *et al.* 2018), physiology (Dodd *et al.* 2005; Resco De Dios *et al.* 2013; Salmela *et al.* 2018; Yarkhunova *et al.* 2018), and shoot architecture (Rubin *et al.* 2018). With regard to plant form or shoot architecture, the number and location of leaves and branches can have significant impacts on fitness (Pigliucci and Schlichting 1996; Rubin *et al.* 2018), and similar to the plasticity of circadian rhythms (Rubin *et al.* 2017), phenotypic expression of these traits differs across environments (Barthélémy and Caraglio 2007; Fournier‐Level *et al.* 2013; Wang *et al.* 2018). The number of both cauline and rosette leaves and branches are, for instance, variable among experimental populations of *A. thaliana* grown under field conditions (Rubin *et al.* 2018), and both branch types show phenotypic correlations with circadian period or phase. Notably, the circadian correlation is in opposing directions for the two branch types; that is, a positive correlation exists between circadian period and cauline branch number, and a negative correlation between circadian period and rosette branch number (Rubin *et al.* 2018). The observed phenotypic correlations elicit questions about the QTL architecture and evolutionary potential of both the circadian clock and correlated branch traits.

Trait covariances can enhance or constrain the expression and evolution of diverse traits, contingent on the direction and strength of selection acting on each trait (Etterson and Shaw 2001). For instance, similarity of QTL regions for two different traits (*e.g.*, circadian period length and branching patterns that determine shoot architecture) may indicate a shared genetic basis, arising from either pleiotropy or physical linkage of multiple causal loci. Similarity of QTL identity and effect across environments (*e.g.*, months of the growing season) for a single trait such as circadian period could indicate that clock responsiveness to one set of conditions (*e.g.*, 12-hr photoperiod and comparatively lower Day/Night temperature differences in early summer) is genetically correlated with the magnitude of potential response to other seasonal conditions *(e.g.*, longer, 16-hr photoperiods and greater Day/Night temperature differences characteristic of mid summer). Whereas, if distinct QTL determine responses to early *vs.*. mid-summer seasonal conditions, then individuals may achieve more nuanced circadian responses to different environmental conditions over the course of the growing season.

Here, we were interested in dissecting the QTL architecture of circadian parameters and aspects of shoot architecture, with the aim of understanding how environmentally responsive QTL were and thus how responsive (or constrained) clock phenotypes are in the wild. We genotyped three sets of *A. thaliana* recombinant inbred lines (RILs) and then phenotyped these lines under early-, mid-, and late-season conditions. The RILs harbor a circadian bioluminescence reporter construct allowing for the quantification of circadian period and phase. We address the following questions: 1) what is the QTL architecture of circadian parameters under field conditions and 2) to what extent do circadian QTL correspond to QTL for plant architecture?

## Methods

### Genetic lines

We present results from three sets of *A. thaliana* RILs that harbor the reporter gene *LUCIFERASE (LUC)* derived from fireflies (*Photinus phralis*) linked to the native circadian gene, *COLD CIRCADIAN RHYTHM RNA BINDING 2 (CCR2)*, allowing for quantification of circadian parameters (Miller *et al.* 1992). The first set of 84 RILs (Ws-2 × C24) is the result of a cross between the natural accessions Ws-2 (♀; Russia) and C24 (♂; Portugal). The second set of 91 RILs (Ws-2 × L*er*) is the result of a cross between Ws-2 (♀; Russia) and L*er* (♂;Poland, formerly Germany; see for example, Zapata *et al.* 2016). Lastly, the third set of 70 RILs (L*er* × Ws-2) are the result of a cross between L*er* (♀) as the maternal parent and Ws-2 (♂) as the paternal parent. For each set, pollen from the paternal parent was transferred to the maternal parent, which harbored the *CCR2: LUC* reporter construct located near the middle of chromosome 3, to generate a heterozygous F1. Each F1 was then backcrossed to the maternal parent and the resulting BC1F1 progeny were selfed through single-seed descent to the BC1F6 generation (Boikoglou 2008; Rubin *et al.* 2017; Rubin *et al.* 2018). Due to the backcrossing generations to different maternal parents, the Ws-2 × L*er vs.* L*er* × Ws-2 populations differ in their cytoplasmic and nuclear genetic composition, both of which may contribute to differences in circadian clock function (Bdolach *et al.* 2018).

### Analyses of circadian rhythms

We measured circadian period and phase on eight replicates per RIL for each of the three RIL populations at three time points (June, July, and September) over the course of the growing season, as described in detail in Rubin *et al.* (2017). In brief, seeds were surface sterilized and pipetted into 96-well white microtiter plates containing Murashige and Skoog media. Following cold stratification of seed, the plates were moved into a Percival PGC-9/2 growth chamber to germinate (12-hour photoperiod, 22°, and 50% relative humidity). After seed germination, seedlings were entrained in the field for 5 days, a period of time sufficient for entrainment to field conditions. Plates were then returned to the lab for bioluminescence imaging. D-luciferin monopotassium salt was added to each well, and the plants were imaged for 4 days using a Hamamatsu ORCA IIER camera (Hamamatsu Photonics C4742-98-24ER). We estimated circadian period and phase using ImagePro/IandA software (Plautz *et al.* 1997; Mcwatters *et al.* 2000; Doyle *et al.* 2002). Circadian period is defined as the average cycle length, and circadian phase is the timing of peak expression relative to dawn on the final day of field entrainment (Rubin *et al.* 2017; Rubin *et al.* 2018).

### Field experiments

Additional replicates of each RIL were screened for a number of life history and morphometric traits. During the first week May 2008, we planted 12 replicates of the Ws-2 × C24 RILs into 5-cm diameter net baskets filled with Sunshine Sungro LP-5 soil and cold-stratified the seed at 4° for four days. The pots were then moved to the greenhouse at the University of Wyoming Agriculture Experimental Station Research and Extension Center Greenhouse Field Plots for seed to germinate. After three weeks, plants were transplanted from the greenhouse to the adjacent field research site (Elevation: 2,226 meters; 41.32° N, 105.6° W) into twelve randomized blocks. The following year, in May 2009, 14 replicates of the L*er* × Ws-2 and Ws-2 × L*er* RIL sets were planted, as described above for 2008 cohort, and transplanted in 14 fully randomized blocks. Plants were misted daily (through germination) and bottom watered manually each day in the greenhouse. Following transplantation into the field, the plants were watered daily by an automatic overhead irrigation system. Detailed planting methods are reported in Rubin *et al.* (2018). Plantings adhered to protocols for transgenic plants described under APHIS BRS notifications 06-100-101n and 12-101-102n, and the field site was reviewed and found compliant by APHIS personnel in three subsequent years (reports 12-037-103n and 14-091-111n).

We measured diverse traits on additional replicates of each RIL. The choice of traits was guided by the known developmental patterns of *A*. *thaliana*. Prior to the transition to reproduction, *A. thaliana* produces a vegetative rosette of leaves (referred to as rosette leaves) where each leaf axil contains a single axillary meristem. At the transition to flowering, the production of rosette leaves ceases, and the leaves produced on the resultant inflorescence stem are referred to as cauline leaves, which also have a single axillary meristem (Grbić and Bleecker 2000). Axillary meristems in cauline and rosette leaf axils can develop into cauline or rosette branches, respectively, or may remain quiescent. Developmentally, cauline axillary meristems develop into branches earlier than those that originate from rosette axillary meristems (Hempel and Feldman 1994). For each plant, we counted the number of rosette leaves at flowering. At senescence, we counted the number of cauline leaves and cauline and rosette branches. We calculated cauline axillary meristem fate (cauline branch number/cauline leaf number) and rosette axillary meristem fate (rosette branch number /rosette leaf number). Note that there was no genetic variation for cauline meristem fate in any of the three populations and therefore, we do not include this trait in the QTL analyses (Rubin *et al.* 2018). We estimated line variance components using restricted maximum likelihood from mixed models, which included RIL and block as random factors (PROC MIXED; SAS 2015). To estimate the mean phenotype of each RIL, we calculated Best Linear Unbiased Predictors (BLUPs) for each RIL within each RIL set (Robinson 1991). Genotypic BLUPs were used to test for bivariate correlations (*r*) between circadian and architectural traits; we refer to these correlations as “phenotypic” throughout the manuscript (PROC CORR; SAS 2015). Notably, circadian data are as presented in quantitative genetic analyses in Rubin *et al.* 2017 and Rubin *et al.* 2018. Here, we present the results of bivariate trait associations for circadian and plant architecture traits and examine the QTL architecture of these traits.

### Marker development and QTL mapping

Markers for each RIL set were developed using a modified genotyping by sequencing approach to identify SNPs. We digested genomic DNA with restriction enzymes (*Eco*RI and *Mse*I), ligated barcoded Illumina adaptors onto the resulting fragments, PCR-amplified fragments with iProof high-fidelity polymerase (Bio-Rad; Hercules, California, USA), reduced the genomic coverage via an agarose gel size-selection step, and pooled samples into a single library for sequencing on the Illumina HiSeq platform (National Center for Genome Resources; Santa Fe, NM, USA). This approach allowed for highly multiplexed sequencing of selected genomic regions from which we identified SNPs that segregated in each RIL set. The Ws-2× C24 RIL set contains 136 genetic markers (SNPs), the Ws-2 × L*er* RIL set contains 111 markers and L*er* × Ws-2 RIL set contains 136 markers.

We were primarily interested in a) obtaining the best estimate of the trait genetic architecture (median number of loci with credible intervals) and b) assessing the potential for genome-wide associations of QTL for pairs of traits, specifically with regards to circadian clock and branching QTL. We therefore used a Bayesian genome-wide regression and variable selection analysis (GEMMA) that estimates the best QTL model when testing for SNP-phenotype associations for each trait (Zhou and Stephens 2014). Further, the crossing design, including backcrossing the F1 to the parent, means that allele frequencies in the RIL populations deviate from those assumed in most marker-based linkage mapping models and exhibit greater genetic structure than traditional mapping populations; as a result mapping in many programs (*e.g.*, QTL Cartographer, R/QTL) are impracticable. GEMMA does not assume a specific segregation pattern at each locus (such as 50/50 of each homozygous class as in a RIL population), and it provides for statistical control of genetic relatedness among individuals within each RIL set using a kinship matrix. GEMMA specifically evaluates multi-SNP models and provides estimates of posterior-inclusion probabilities (PIP) for each marker as well as a model-averaged percent variance explained for the genetic architecture of each trait. Notably, because the estimation procedure aims primarily to identify the best model of the genetic architecture, different SNPs with low explanatory power may be identified in each MCMC step, and therefore the QTL localization is precise only for SNPs that explain a large proportion of the phenotypic variation.

Markov chain Monte Carlo (MCMC) samples were initiated with 50,000 burn-in steps, followed by 20,000,000 sampling interactions with every 200^th^ step retained (*i.e.*, analyses are based on 100,000 MCMC samples). Here, we report the estimated numbers of SNPs with significant phenotype associations and percent variance explained (median and 95% credible interval, equal tail probability interval (ETPI)) and compare the SNP PIP scores across traits for each of the three RIL sets. More specifically, following the genome-wide scan identifying the best-fit model for median QTL number, we tested for correlations between PIP values for all SNPs to test for bivariate trait associations at the QTL level.

Using the estimates of the median SNP number for each trait, we identified the respective number of SNPs with the highest PIP values. We then identified SNPs that jointly affect both period or phase and a second trait were further examined for trait associations. Specifically, we tested for phenotypic differences across genotypic classes using a linear model that contained the genotypic class of the SNP of interest as a fixed effect. From this model, we estimated least-squared means for each genotypic class for each trait. Starting at the focal SNP, we permuted through linear models that contained the next upstream or downstream SNP(moving away from the target SNP in a stepwise fashion where each model contained a single SNP) until we identified the closest upstream and downstream SNP that was no longer associated with the trait to operationally define a confidence interval and a window in the genome to investigate for potential candidate genes with known function in aspects of the circadian clock and plant architecture (Lamesch *et al.* 2011).

### Data accessibility

Data used to conduct statistical analysis are publicly available at https://doi.org/10.25387/g3.7182296. Supplemental material available at Figshare: https://doi.org/10.25387/g3.7182296.

## Results

In the Ws-2 × C24 RIL set, the median number of QTL detected across all traits ranged from 4 to 14 (Supplementary Figure 1). The credible intervals for these estimates in most cases included much higher QTL numbers, suggesting that most traits could have many more significant QTL (Table 1). While for some traits the credible interval contained an estimate of zero QTL, it is unlikely that no QTL exist given all traits exhibited significant genetic variances; instead, QTL effects were likely below the detection level of the mapping population. When summed, all mapped QTL for a given trait explained between 13 to 80% of the total genotypic variation, with credible intervals suggesting that the median was often closer to the high end for the total percent variance explained (Table 1). For the Ws-2 × L*er* and L*er* × Ws-2 RIL sets, the median number of QTL mapped across all traits ranged from 6 to 14 and 5 to 13, respectively, and explained 5 to 58% of the genotypic variation in a given trait (Table 1); patterns for the credible intervals were similar to those described for Ws-2 × C24, including high estimated QTL numbers as well as estimates of zero QTL. Notably, the top two to four scoring SNPs for period and phase in each RIL set explained a large proportion of the variance in the median QTL model for each trait (compare median PVE in Table 1 for July period and phase to the sum of individual SNP *R*^2^-values for the same trait in Table 2; the *R*^2^ values for individual SNPs presented in Table 2 may be overestimates as the ANOVA models estimating percent variance explained do not account for genetic variation segregating elsewhere in the genome or kinship). These results are consistent with a genetic architecture with a few QTL of moderate-large effect and many additional QTL of small effect.

**Table 1:** Median number of SNPs included in the model and median percent variance explained (PVE) by the model for each trait for the *Arabidopsis thaliana* Ws-2 × C24, Ws-2 × L*er* and L*er* × Ws-2 RIL sets. 95% credible intervals around the median are shown in parentheses

	Ws-2 × C24 RIL set		Ws-2 × L*er* RIL set		L*er* × Ws-2 RIL set	
	Median SNP	Median PVE	Median SNP	Median PVE	Median SNP	Median PVE
June Circadian Period	9 (0-62)	0.47 (0.25-0.67)	7 (0-61)	0.14 (0.01-0.42)	6 (0-61)	0.14 (0.01-0.45)
July Circadian Period	10 (0-61)	0.36 (0.11-0.66)	13 (0-64)	0.54 (0.30-0.75)	7 (0-60)	0.26 (0.06-0.52)
Sept. Circadian Period	7 (0-61)	0.09 (0-0.30)	7 (0-61)	0.06 (0-0.24)	8 (0-61)	0.38 (0.09-0.69)
June Circadian Phase	12 (0-63)	0.48 (0.25-0.66)	6 (0-60)	0.07 (0-0.28)	9 (0-62)	0.25 (0.03-0.55)
July Circadian Phase	8 (1-57)	0.55 (0.22-0.82)	10 (0-63)	0.23 (0.03-0.49)	5 (0-60)	0.06 (0.00-0.27)
Sept. Circadian Phase	8 (0-62)	0.13 (0-0.35)	6 (0-61)	0.05 (0-0.22)	7 (0-61)	0.12 (0.01-0.39)
Cauline Leaf Number	6 (0-60)	0.49 (0.28-0.68)	7 (0-60)	0.20 (0.02-0.48)	12 (0-64)	0.49 (0.19-0.74)
Cauline Branches	6 (0-59)	0.52 (0.31-0.71)	9 (0-62)	0.17 (0.01-0.43)	12 (0-63)	0.38 (0.12-0.63)
Rosette Leaf Number	5 (2-43)	0.78 (0.51-0.92)	6 (1-57)	0.36 (0.14-0.59)	13 (0-63)	0.61 (0.35-0.82)
Rosette Branches	14 (0-64)	0.65 (0.42-0.83)	14 (0-63)	0.49 (0.24-0.71)	7 (1-59)	0.53 (0.29-0.73)
Rosette Meristem Fate	7 (0-61)	0.27 (0.03-0.57)	9 (0-62)	0.19 (0.02-0.43)	10 (0-62)	0.53 (0.31-0.72)

**Table 2:** General linear models for the top scoring SNPs for circadian period and phase measured in July for the *Arabidopsis thaliana* Ws-2 × C24 (A), Ws-2 × L*er* (B) and L*er* × Ws-2 (C) RIL sets. Chromosome and nucleotide position for each SNP are provided. R^2^ for each SNP as estimated from ANOVA models and phenotypic means and standard errors for genotypic classes are shown

							Genotypic Mean ± SE.	
	Trait	Chr.	Nucleotide	*F*-value	*P*-value	*R*^2^	Ws-2/Ws-2	C24/C24
A. Ws-2 × C24	Period	2	14,500,577	12.71	0.0006	0.14	23.40 ± 0.11	23.90 ± 0.08
		3	5,551,187	4.56	0.036	0.06	24.37 ± 0.30	23.70 ± 0.07
		3	9,377,864	4.00	0.0492	0.05	23.42 ± 0.17	23.80 ± 0.08
		5	669,813	6.58	0.0124	0.08	24.06 ± 0.14	23.65 ± 0.08
	Phase	1	27,046,266	9.27	0.0013	0.13	14.43 ± 0.16	15.13 ± 0.14
		3	5,551,187	4.91	0.0296	0.06	15.83 ± 0.48	14.74 ± 0.11
		3	9,377,864	7.45	0.0079	0.09	14.17 ± 0.26	14.95 ± 0.12
		3	18,651,928	12.97	0.0006	0.15	14.39 ± 0.15	15.14 ± 0.14
							Genotypic Mean ± SE.	
	Trait	Chr.	Nucleotide	*F*-value	*P*-value	*R*^2^	Ws-2/Ws-2	L*er*/L*er*
B. Ws-2 × L*er*	Period	3	849,706	18.71	0.0001	0.20	23.68 ± 0.13	22.92 ± 0.11
		3	8,816,904	4.98	0.0286	0.06	22.67 ± 0.27	23.31 ± 0.10
		5	1,335,242	26.06	0.0001	0.26	24.13 ± 0.19	23.03 ± 0.09
	Phase	1	5,689,169	3.15	0.08	0.04	11.67 ± 0.38	10.95 ± 0.14
		2	17,179,798	8.47	0.0047	0.10	10.12 ± 0.34	11.18 ± 0.14
		3	849,706	5.23	0.0250	0.07	10.78 ± 0.17	11.38 ± 0.20
							Genotypic Mean ± SE.	
	Trait	Chr.	Nucleotide	*F*-value	*P*-value	*R*^2^	L*er*/L*er*	Ws-2/Ws-2
C. L*er* × Ws-2	Period	3	849,706	14.10	0.0004	0.18	23.31 ± 0.42	20.45 ± 0.64
		5	9,524,947	8.22	0.0056	0.11	22.96 ± 0.40	20.34 ± 0.82
		5	24,941,285	17.48	0.0001	0.22	21.66 ± 0.39	24.99 ± 0.70
	Phase	1	18,082,872	11.55	0.0012	0.15	19.08 ± 0.22	14.88 ± 1.22
		5	24,941,285	6.91	0.0107	0.10	18.57 ± 0.25	19.94 ± 0.45

Significant within-month correlations between period and phase were observed at the genotypic and QTL level in all three RIL sets; for instance, SNP PIP values for circadian period length in July were significantly correlated with phase in July (Table 3, Supplementary Figure 2B). Within each month, one of the top scoring QTL affected both period and phase (Table 2). Further, based on visual inspection of the bivariate SNP associations, multiple QTL appeared to contribute to the period-phase correlation in July, while a lesser number of contributing QTL are observed in September (cf Supplementary Figure 2B *vs.*. 2C).

**Table 3:** Pearson correlations between circadian period and phase estimates and SNP posterior inclusion probabilities (PIPs) across the growing season (June, July and September (Sept.)) for the *Arabidopsis thaliana* Ws-2 × C24 (top value), Ws-2 × L*er* (middle value) and L*er* × Ws-2 (bottom value) RIL sets. Correlations in gray were originally reported in Rubin *et al.* 2017. *** *P* < 0.001, ** *P* < 0.01, * *P* < 0.05

Trait Pair	*r*_phenotypic_	*r*_PIP_
June Period and Phase	0.49***	0.13
	0.11	0.49***
	0.28*	−0.05
July Period and Phase	0.83***	0.80***
	0.65***	0.48***
	0.31**	0.36***
Sept. Period and Phase	0.71***	0.56***
	0.31**	0.96***
	−0.16	0.17*
June and July Period	0.55***	0.30***
	0.39**	0.01
	−0.20	0.16
June and Sept. Period	0.10	−0.01
	0.43***	0.02
	−0.03	−0.06
July and Sept. Period	0.06	−0.08
	0.42***	−0.05
	0.04	−0.05
June and July Phase	0.20*	−0.05
	−0.18	−0.01
	−0.17	−0.03
June and Sept. Phase	0.18	0.17*
	−0.05	−0.09
	0.13	−0.01
July and Sept. Phase	−0.08	−0.05
	0.06	−0.03
	−0.12	0.02

The strength of within-trait associations across months differed for circadian period *vs.*. phase. Overall, a greater number of phenotypic correlations were observed across months for period (4 out of 9 possible instances) than for circadian phase (1 of 9; Table 3), and the phenotypic correlations for period were of greater magnitude. In the Ws-2 × L*er* RIL set, monthly period estimates were correlated across all three months (*i.e.*, June with July, June with September and July with September), whereas the bivariate correlations between monthly period values were non-significant in all but one case within the Ws-2 × C24 and L*er* × Ws-2 RIL sets (Table 3). Despite strong phenotypic correlations, at the QTL level there was little correspondence between the SNPs with high posterior inclusion probabilities for one month with those of a second month for either circadian period and phase (see *r*_PIP,_ Table 3).

**Figure 1:**
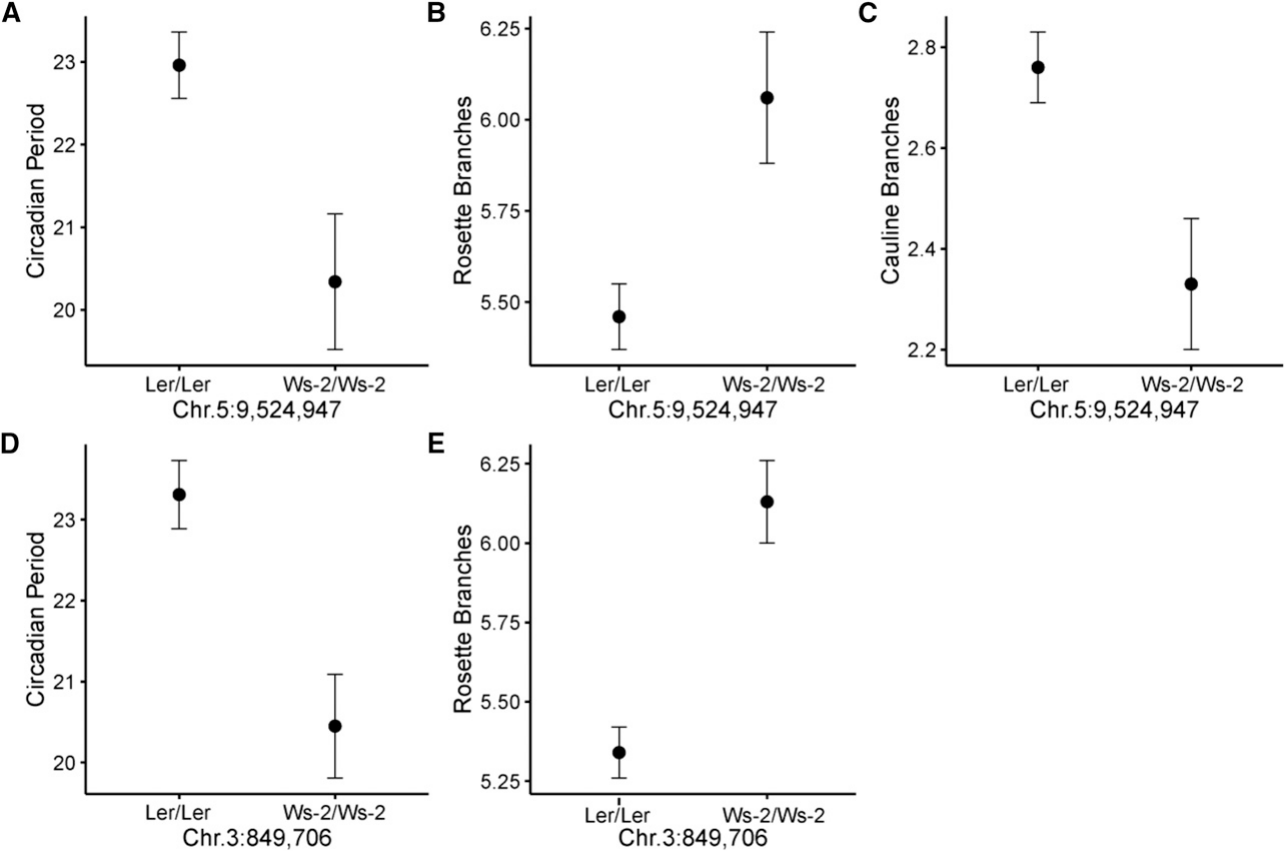
Phenotypic means and standard errors of the *Arabidopsis thaliana* L*er* × Ws-2 RIL set for circadian period (A), rosette branch number (B), and cauline branch number (C) for the two allelic classes (*Ler*/*Ler vs.* Ws-2/Ws-2) at SNP Chr.5:9,524,947 and circadian period (D), and rosette branch number (E) at SNP Chr3:849,706.

Circadian period and phase were correlated with other measured phenotypes, and these associations varied among mapping populations (Supplementary Table 1). QTL for circadian traits were shared with both cauline and rosette branching in at least one mapping population (Supplementary Table 2). A SNP on chromosome 5 (nucleotide 9,524,947) affected circadian period, cauline and rosette branch number in the L*er* × Ws-2 RIL set. Specifically, RILs homozygous for the L*er* SNP state had longer period lengths (*F*_1_ = 8.22, *P* = 0.0056, *R^2^* = 0.11; Figure 1A), fewer rosette branches (*F*_1_ = 9.33, *P* = 0.0033, *R^2^* = 0.13; Figure 1B) and more cauline branches (*F*_1_ = 8.49, *P* = 0.0049, *R^2^* = 0.12; Figure 1C). We also identified a second SNP that contributed to circadian and architectural traits in a consistent manner on the top of chromosome 3 (nucleotide 846,706), specifically, the L*er* SNP was associated with lengthened circadian period cycles (*F*_1_ = 14.10, *P* = 0.0004, *R^2^* = 0.18; Figure 1D) and reduced rosette branching (*F*_1_ = 26.81, *P* < 0.0001, *R^2^* = 0.29; Figure 1E). Cauline leaf number and cauline branch number were highly positively correlated at the phenotypic level (correlation coefficients greater than 0.80 in all three RIL sets, Supplementary Table 1) and also had a high degree of overlapping QTL (*r* greater than 0.53, Supplementary Table 2). A similar positive association was observed in two of the three RIL sets (Ws-2 × C24 and Ws-2 × L*er*) for rosette leaf and branch number. Notably, associations between cauline and rosette leaf number as well as between cauline and rosette branch number were inconsistent at both the phenotypic and QTL level (Supplementary Table 1 and 2). Specifically, the correlation between cauline and rosette leaf number was significant in two of the three RIL sets at the phenotypic level (Ws-2 × C24 and L*er* × Ws-2; Supplementary Table 1) but only in the Ws-2 × C24 RIL set at the QTL level (Supplementary Table 2). Similarly, the association between cauline and rosette branch number was significant at the phenotypic and QTL level only in the Ws-2 × C24 RIL set (Supplementary Table 1 and 2). Collectively, the significant associations where *r* < 1 in some RIL populations as well as non-significant associations in other RIL populations suggest a partially independent genetic basis for the expression of cauline *vs.*. rosette leaf number as well as cauline *vs.*. rosette branch number (Supplementary Table 1 and 2).

To investigate whether the associations between circadian period and cauline and rosette branching in the L*er* × Ws-2 RIL set potentially resulted from loci with pleiotropic effects, we queried the two genomic regions mentioned above (chromosome 3: nucleotide 846,706 and chromosome 5: nucleotide 9,524,947) for genes with known effects on the circadian clock and branching patterns. For the SNP located on chromosome 3, we identified two separate candidate genes: *AtMBD9* (AT3G01460) for rosette branch number and *WNK1* (AT3G04910) for circadian period. A single strong candidate gene, *TPR3* (AT5G27030), emerged from the genomic region near the SNP on chromosome 5 for circadian period, cauline and rosette branch number.

## Discussion

The natural environment that plants experience over the course of their lifespan is dynamic, and accurate perception and response to the changing environment is adaptive (Ouyang *et al.* 1998; Dodd *et al.* 2005; Yerushalmi *et al.* 2011; Greenham *et al.* 2017; Rubin *et al.* 2017; Rubin *et al.* 2018). The circadian clock comprises one developmental mechanism that facilitates coordination of the internal biological rhythms of an organism to the external environment. Experiments in controlled conditions where light and temperature were manipulated demonstrate that the circadian clock responds, or entrains, to varying environments (Somers *et al.* 1998; Edwards *et al.* 2005; Hotta *et al.* 2007; Lou *et al.* 2011; Mcwatters and Devlin 2011; Anwer *et al.* 2014; De Montaigu *et al.* 2015; Inoue *et al.* 2017). However, plasticity in response to a single environmental input may differ from clock phenotypes observed in natural conditions where many environmental inputs vary simultaneously. In order to understand the adaptive potential of the circadian clock it is important to understand how the genetic architecture of the clock varies and may therefore constrain or facilitate an organism’s ability to produce multiple circadian phenotypes over its lifetime.

A shared genomic architecture may reflect functional relationships among traits (Armbruster and Schwaegerle 1996). For instance, circadian period and phase are typically anticipated to have a positive correlation, because phase is mathematically related to the circadian period, that is, delayed phase is anticipated in genotypes with longer periodicity. We find correspondingly that period and phase are positively correlated at both the phenotypic and QTL level. However, phenotypic correlations where *r* < 1and the lack of QTL synteny across months of the growing season, *e.g.*, non-significant or non-perfect correlations among SNPs for phase in July *vs.*. September, may allow selection to act on clock parameters across variable environments somewhat independently and thus allow for adaptive circadian timing across the growing season and over an organism’s lifespan. This may be especially important for annual plants, like *A. thaliana*, where matching the biological activities to external cycles is especially important due to their short lifespan. This finding is consistent with previous work under controlled conditions that demonstrated that the genetic architecture of circadian parameters is a composite of overlapping QTL that are detected across many environmental conditions and others that are environment-specific (Michael *et al.* 2003; Edwards *et al.* 2005; Boikoglou *et al.* 2011; Lou *et al.* 2011; Müller *et al.* 2018), and that circadian phase is responsive across the growing season (Matsuzaki *et al.* 2015).

Plant architecture can be highly variable and aspects of plant form, specifically branching patterns, are important determinants of fitness (Fournier‐Level *et al.* 2013; Wolfe and Tonsor 2014). Recent work identified a novel association between the circadian clock and branching patterns under field conditions (Rubin *et al.* 2018). Therefore, we were specifically interested in testing if clock associations identified in structural equation modeling and supported by clock mutant screens (Rubin *et al.* 2018) were identified at the QTL level for rosette branch number. In the L*er* × Ws-2 RIL set, which showed a significant clock-rosette branch association in prior structural equation modeling, there was significant overlap between QTL for the two traits, where SNPs with the highest likelihood of contributing to circadian period also affected rosette branching. Visual inspection of these bivariate plots suggests they are driven by a small number of loci (or multiple loci in tight physical linkage), particularly in June and September, with somewhat more contributing QTL in July (Supplementary Figure 3).

We next considered potential candidate genes in the regions where SNP variation affected the expression of both circadian period and plant architecture. On the top of chromosome 3, the strongest candidate was *AtMBD9* (AT3G01460), which is a transcriptional regulator. Mutants in this gene produce more branches compared to wild-type plants, despite having fewer leaves (and therefore meristems that give rise to branches) (Peng *et al.* 2006). However, this particular gene does not show circadian expression patterns (Covington *et al.* 2008). This region on chromosome 3 has also been implicated in the expression of circadian traits (De Montaigu *et al.* 2015) with one strong candidate being the protein kinase, *WNK1* (AT3G04910), which acts in signal transduction (Murakami-Kojima *et al.* 2002; Wang *et al.* 2008; Kumar *et al.* 2011). Therefore, it is likely the contribution of this genomic region to the expression of circadian period and branching patterns results from separate, linked genes. Alternatively, for the QTL on Chromosome 5, one strong candidate gene, with pleiotropic effects on circadian period and branching, is *TOPLESS-RELATED 3* or *TPR3* (AT5G27030), which directly or indirectly interacts with genes in the core oscillator (*PRR* gene family, *CCA1* and *LHY*) of the circadian clock and regulates many developmental processes, including meristem specification (Long *et al.* 2006; Wang *et al.* 2013). In cases where a single gene is pleiotropic in effect or two adjacent genes are in tight linkage, allele frequency changes at the contributing circadian locus may could lead to a correlated response in branching.

In some cases, a single individual can produce developmentally similar structures on different parts of the plant. For example, in *A. thaliana*, a leaf can be part of the vegetative rosette or be positioned on the inflorescence (cauline leaf). Likewise, branches can be produced from leaf axils in the rosette or on the inflorescence (cauline branch). The degree to which pairs of leaf or branch traits can evolve independently relies, at least in part, by the degree of overlap in their genetic architectures. Prior studies have found that the genetic basis of cauline *vs.*. rosette leaf number and cauline *vs.*. rosette branch number is a composite of QTL that affect both traits and QTL that affect only one (Ungerer *et al.* 2002; Keurentjes *et al.* 2007). We observe that phenotypic correlations are moderate between leaf or branch types (that is, between cauline *vs.*. rosette leaves or between cauline *vs.*. rosette branches). Likewise, QTL are mostly specific to one leaf or branch type, that is, the underlying QTL that determine expression of cauline leaf number are largely (although not entirely) different from those that determine rosette leaf number, consistent with phenotypic correlations greater than 0 but less than 1. In sum, the QTL results for different leaf and branch types suggest that selection can refine each phenotype independently, allowing for the adaptive expression and evolution of both.

The circadian system is dynamic and highly responsive to changing external conditions, which may allow organisms to adaptively match their internal biological rhythms to their local conditions on a small, refined timescale (*i.e.*, day to day) over the course of their lifespan. We have demonstrated that the genetic architecture of clock expression across months is independent, which may facilitate optimal circadian timing across the growing season. Moreover, the time scale in which the genetic architectures vary may be more refined than the monthly scale measured here. The fitness consequences or benefits to plasticity in the circadian clock over an organism’s lifespan has yet to be investigated. Yet, it is notable that genotypes found at higher latitudes show greater clock plasticity to photoperiod than do genotypes derived from lower latitudes where environmental inputs show less intra-annual variation (De Montaigu and Coupland 2017), suggesting that clock sensitivity may evolve in adaptive manner. Further analysis of clock phenotypes and the genetic underpinnings will provide important insights into plant population success under changing environments.
